# Cetuximab modifies the release and protein content of tumor microvesicles from head and neck squamous cell carcinoma cells: What are the consequences on endothelial cells?

**DOI:** 10.1002/ccs3.70026

**Published:** 2025-08-04

**Authors:** Valérie Jouan‐Hureaux, Béatrice Faivre, Cédric Boura

**Affiliations:** ^1^ Université de Lorraine CNRS CRAN UMR 7039 Nancy France; ^2^ Faculté de Pharmacie Université de Lorraine Nancy France

**Keywords:** angiogenesis, cetuximab, endothelial cells, HNSCC, microvesicles

## Abstract

Cancer cells can release extracellular vesicles (EVs) of different sizes under stress conditions. Among the EVs, microvesicles (MVs), which have a size between 50 and 1000 nm, are bounded by a membrane lipid bilayer, exhibit proteins at their surface, and enclose some soluble proteins. MVs can interact with surrounding cells present in the tumor microenvironment to favor tumor resistance. Indeed, they can transport some oncoproteins such as epidermal growth factor receptor (EGFR) and modify phenotype of endothelial cells (ECs). Even if their role in cell communication is well established, the understanding of anticancer treatments on their release and their protein content change are of particular importance. In this work, we showed that head and neck squamous cell carcinoma (HNSCC) cells exposed to cetuximab, monoclonal antibody targeting EGFR, can modulate EGFR expression of MVs. Moreover, this work emphasizes the effect of cetuximab on the shedding and content of MVs by HNSCC cells as well as their interaction with ECs. Consequently, MVs can be used as surrogate markers for predicting the efficacy of anti‐EGFR therapies. Finally, the release of MVs after treatment must be envisaged as a resistance mechanism and must be considered in the future to evaluate the effect of therapy on the tumor microenvironment.

## Graphical abstract

The legend of the graphical summary is as follows: “Microvesicles can interact with surrounding cells present in the tumor microenvironment or at a distance to favor tumor progression or resistance to treatments, and microvesicles are involved in the change of stromal cell characteristics of the tumor microenvironment. The protein content of microvesicles after cetuximab exposition moves toward proteolytic and angiogenic activities. Tumor microvesicles of HNSCC cells express EGFR which are decreased after cetuximab exposition, and microvesicles can be used as surrogate markers for predicting the efficacy response of anti‐EGFR therapies.”

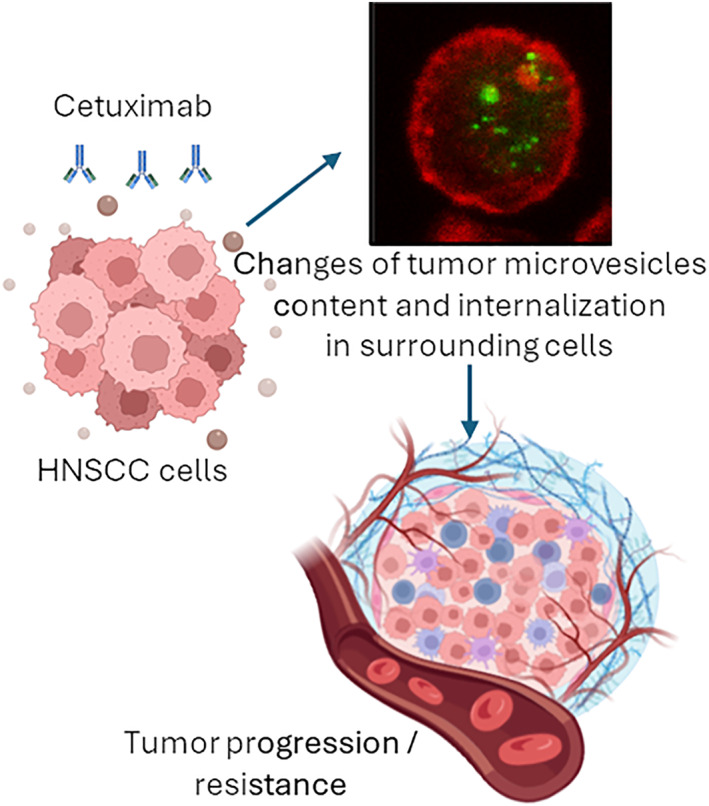

## 1 INTRODUCTION

A mechanism based on the release and uptake of membrane‐bound vesicle, known as extracellular vesicles (EVs), has emerged as a major way of intercellular communication.^1^ EVs can mediate communication both locally and at distant sites. These small, spherical membrane fragments are released from the plasma membrane of eukaryotic cells during apoptosis or following cellular activation under various stress conditions.^2^ EVs are bounded by a lipid bilayer and are typically classified in two categories based on their size and biogenesis: exosomes, which are smaller (diameter 30–150 nm) and originate from multivesicular bodies that are released upon fusion with the plasma membrane and microvesicles (MVs), which range from 50 to 1000 nm and are formed by the outward budding and fission of the plasma membrane. It has been demonstrated that during oncogenic transformation, tumor cells acquired the ability to constitutively release MVs. These MVs can directly interact with surrounding stromal cells present in the tumor microenvironment or at distant sites, thereby promoting tumor progression.^3^, ^4^ MVs can display a variety of surface proteins (such as MMP, PAI‐1, uPA…) and encapsulate soluble factors such as vascular endothelial growth factor (VEGF). Although the biological functions of exosomes have been extensively studied,^5^ the role of MVs in cancer progression and particularly in tumor‐induced angiogenesis remains less well characterized.

Head and neck squamous cell carcinoma (HNSCC) is the sixth most common cancer worldwide, accounting for approximately 890,000 new cases per year and 450,000 deaths annually as of 2018.^6^ Overexpression of the epidermal growth factor receptor (EGFR) is detected in 90%–100% of HNSCC cases and is associated with poor prognostic.^7^ For several years, cetuximab, a monoclonal antibody directed against EGFR, has been used in combination with radiotherapy for the treatment of HNSCC. However, resistance to cetuximab has increasingly been reported.^8^ Although this resistance has been partly attributed to hyperactivation of PI3K/AKT and RAS/MAPK/ERK signaling pathways,^9^ a number of diverse mechanisms of acquired resistance have been identified in HNSCC patients.^10^, ^11^ These mechanisms likely reflect the inter‐patient heterogeneity as well as the influence of the tumor microenvironment and tumor anatomic site.^12^ In 2009, Bergmann et al. demonstrated the presence of MVs in sera of patients with HNSCC and their potential role in tumor progression.^13^ Recently, Fujiwara et al. reported that exosomes secreted by oral squamous cell carcinoma are inhibited by cetuximab, which disrupts EGF‐induced epithelial to mesenchymal transition (EMT).^14^, ^15^ Although existing data on MVs in HNSCC are promising, a more detailed analysis of their molecular content may reveal novel biomarkers and help to elucidate mechanisms of tumor progression and therapeutic resistance, particularly during anti‐EGFR therapy. To our knowledge, this is the first study to evaluate the effects of cetuximab on the release and the molecular content of MVs in HNSCC as well as the capacity of these MVs to interact with endothelial cells (ECs).

## 2 MATERIAL AND METHODS

### 2.1 Endothelial cell culture

Umbilical cords were cut after delivery in compliance with relevant laws in respect with consent of women. Human umbilical vein ECs (HUVECs) were collected from umbilical cords and cultured until Passage 5 in “HUVEC medium” consisting in 50% M199 (GibcoBRL) and 50% RPMI 1640 (v/v) (Sigma) supplemented with 20% heat inactivated human AB serum (EFS, Nancy), 2 mM L‐glutamine (GibcoBRL), 100 U/mL penicillin (GibcoBRL), 100 μg/mL streptomycin (GibcoBRL), 2.5 μg/mL amphothericin B (GibcoBRL), and 20 mM HEPES (Sigma).^16^

### 2.2 Microvesicles preparation

HNSCC cell lines isolated from a patient prior to treatments was obtained from Dr J.L. Fischel (Center Antoine Lacassagne, Nice, France) and purchase from ATCC (Cal27: human tongue squamous cell carcinoma, CRL‐2095, ATCC; FaDu: human hypopharynx squamous cell carcinoma, HTB‐43, ATCC) were cultured in RPMI 1640 medium (GibcoBRL, France) supplemented with 10% heat inactivated fetal calf serum (FCS) (Dutscher, France), 2 mM L‐glutamine (GibcoBRL), 100 U/mL penicillin (GibcoBRL, France) and 100 μg/mL streptomycin (GibcoBRL, France). At 50% confluence, cultures of Cal27 or FaDu were exposed to 2% FCS MVs free with (C20) or without (NT) 20 μg/mL cetuximab (Erbitux^®^, Merck Serono) for 48 h. FCS MV‐free was obtained after two centrifugation steps at 15,000 G during 1h30.

MVs were collected after the centrifugation of conditioned media (CM) at 2600 G (two times) (MVs obtained from Cal27: MV Cal27; MV obtained from FaDu: MV FaDu) and used for flow cytometry analysis. For western blot and arrays analysis, MVs were concentrated and washed three times with PBS using ultrafiltration centrifugal filter devices (Pierce^®^ concentrator 150 KDa, Thermo Scientific). Protein content was evaluated using a bicinchoninic acid assay (BCA^TM^ protein assay kit, Thermo scientific). MV‐DiO was obtained from cultures of HNSCC labeled with 2 μM DiO (Fluoprobes, Molecular Probes, Interchim) during 15 min and washed three times with PBS before seeding. Control CM, control MV, and control DiO media were collected as described for CM, MV, or MV‐DiO preparation, respectively, in which cells were replaced by PBS. All of these preparations were used freshly to preserve their integrity.

### 2.3 Scanning electron microscopy

HNSCC cells were cultivated onto plastic coverslips (Thermanox^®^ 13 mm, NUNC, Thermofisher Scientific) and exposed with (C20) or without (NT) 20 μg/mL cetuximab during 48 h. After three washes with PBS, cells were prepared for scanning electron microscopy analysis: washes with PBS, fixation overnight in 2% glutaraldehyde in cacodylate buffer, washes with ultrapure water and deshydratation with ethanol before metallization (gold/palladium) using a sputter coater Polaron Range SC7640 ThermoVG Scientific apparatus. The morphology of cells were analyzed using a Cambridge Stereoscan S240 apparatus of the platform “Service commun de microscopie” of “Université de Lorraine” (Nancy). Photomicrographs were taken under an acceleration voltage of 20 kV.

### 2.4 Flow cytometry analysis

Analysis of MVs was performed using the Navios cytometer apparatus (Beckman Coulter) checked daily with calibrated fluorescent beads (Flow‐Check® Fluorospheres, Beckman Coulter). A mixture of size calibration beads (diameter: 0.5, 0.9, and 3 μm) (Megamix^®^, Diagnostica Stago) was used to define the population of MVs: the upper limit of the gate was done by beads of 0.9 μm diameter. The release of MVs by HNSCC was quantified by the number of events counted for 300 s at the flow rate of 0.5 μL/sec and reported per unit of the volume. Annexin‐V‐APC (BD Biosciences), anti‐human EGFR‐FITC (AbD serotec), EpCAM‐PerCP‐Cy5.5 (BD Biosciences) or isotype control binding of MVs was evaluated after 30 min of incubation in annexinV binding buffer (BD Biosciences).

### 2.5 Protein analysis of MVs using human angiogenesis array and human soluble receptor array kit

Sub‐confluent cultures of HUVEC were incubated with control, CM, or MV media during 24 h. Then, HUVECs were lysed during 30 min and protein extracts were collected and kept frozen at −20°C until analyzed. Protein concentration was quantified using the BCA^TM^ protein assay (Thermo Scientific). Three hundred microgram of proteins from MV lysate were deposed onto nitrocellulose membranes and incubated overnight at +4°C and used as indicated by the manufacturer. Unbound materials were eliminated by washing and bound materials were detected using antibody conjugated with horseradish peroxidase and ECL prime western blotting detection reagent kit (Amersham, England). Detection was performed with a chemiluminescent detection system (Las imager 4000) and analyzed by an image analysis software (Multigauge®).

### 2.6 Fluorescence microscopy

Subconfluent cultures of HUVEC were incubated with control, CM, or CM‐DiO‐MV media during 24 h. HUVECs were then removed from their support by trypsinization (Trypsin, Sigma‐Aldrich) and stained 15 min with anti‐PECAM‐APC antibody (CD31, e‐Bioscience) for cytoplasmic membrane and/or 5 min with DAPI for the nucleus (Molecular probes) after the fixation of cells (Intrastain A permeabilization kit, Dako). Fluorescence of HUVECs was then analyzed using SP2 confocal microscope (Leica Zeiss) and ImageStream^X®^ apparatus (Amnis Corporation, Merck Millipore).

For confocal microscopy analysis, cells were plated 5 min at 500 G onto glass slides using cytospin autosmear CF‐12DE device (Sakura Finetechnical Co) before analysis in Z‐series of 20 pictures (voxel size: 91.55 × 91.55 nm, speed 400 Hz, pinhole 1, picture size 512 × 512). Images were done after 3D reconstruction and 2D projection with Image J software.

For image flow cytometry analysis, focus and single cells were selected using gradient Root Mean Square and area ratio function of the analysis software of the ImageStream® (IDEAS, Amnis Corporation). The percentage of cells with DiO‐positive stain was evaluated using the biparametric histogram [[Intensity, M02, CH02] = *f* [modulation, MO2, CHO2]] feature of IDEAS. Then, the number of spots per cell was evaluated using “Peak(Spot (Intensity [M02, CH02, 50‐4095], CH02, Bright 3,1) CH02, Bright 3)” mask and count spot feature of the software.

### 2.7 Endothelial cell network formation

EC network formation was analyzed as previously described (Jouan‐Hureaux et al., Microvasc Res 2012). Briefly, GFP‐HUVEC (Essen Bioscience) was plated on Matrigel^®^ precoated wells (BD Biosciences) and incubated with control, CM, or MV media during 24 h. After fixation with PAF 4%, photomicrographs were taken (Nikon AZ100, Digital Sight DS‐Qi1Mc camera, Nikon). EC density was calculated with NIS‐Elements Software (Nikon) and represented as the area fraction in relation to the whole field. The total additive length of all cellular structures including all branches and the number of junction were quantified with Angiosys software (TCS Cellworks).

### 2.8 Statistical analysis

All results were given as mean ± standard deviation. Differences among groups were tested using variance analysis (ANOVA) followed by the Bonferroni/Dunn test (Statview software 5.0). Values of *p* < 0.05 were considered as statistically significant.

## 3 RESULTS

### 3.1 Effect of cetuximab on MVs release by HNSCC cells

**FIGURE 1:**
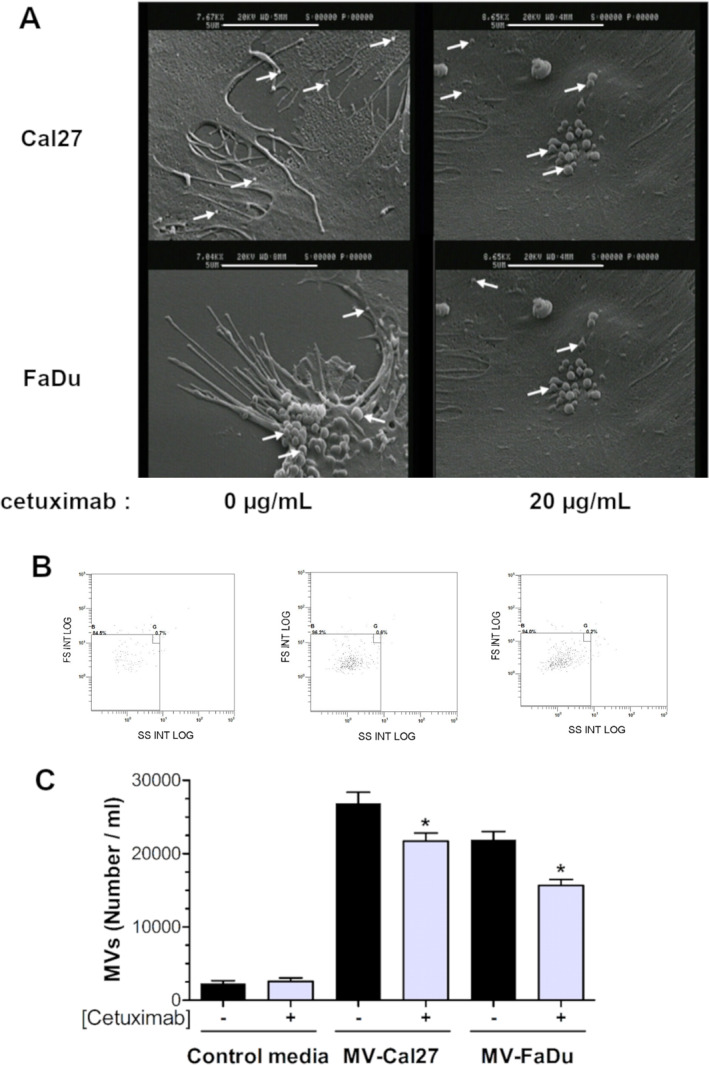
Characterization of microvesicles (MVs) released by head and neck squamous cell carcinoma (HNSCC) cells. HNSCC cells were cultivated in the presence or absence of cetuximab (0 or 20 μg/mL) in 2% fetal calf serum (depleted from MVs) during 48 h. The scanning electron microscopy observation of the morphology of the HNSCC surface (A). White arrows indicate the presence of MVs at the HNSCC membrane surface (5 μm white scale bars are shown on each image). MVs released in culture media were counted using flow cytometry. Megamix® beads (B) and reported as number of MVs per mL of media (C) (*n* = 4 in duplicate). Differences are considered as significant when *p* < 0.05 using the Bonferroni/Dunn test. * versus same group without cetuximab.

MVs released by HNSCC cultured with or without cetuximab were observed using scanning electron microscopy. Photomicrographs revealed the presence of small, regularly shaped buds ranging in size from 0.13 to 1.04 μm in diameter, consistent with the expected MVs size range (Figure 1A). MVs appeared both on cytoplasmic extensions on the main cell body (see white arrows). Cetuximab treatment did not alter the localization of the relapse or the morphology of MVs.

To evaluate MVs release and the effect of cetuximab on MVs shedding, we quantified MVs in CM by flow cytometry using Megamix beads as a normalization tool. A significant number of MVs were detected in CM from Cal27 or FaDu (referred to as MV‐Cal27 or MV‐FaDu, respectively) (Figure 1B), with values approximatively 10‐fold higher than those from control media (Figure 1C). Cetuximab exposure led to a reduction in the number of MVs by these two cell lines: −19% for Cal27; −32% for FaDu. The MVs size distribution was monodisperse with a single peak regardless of the cellular origin (Supporting Information S1: S1). The median diameter of MVs (approximately 0.53 μm) remained unchanged upon cetuximab treatment.

### 3.2 EGFR expression on MVs after cetuximab exposure

**FIGURE 2:**
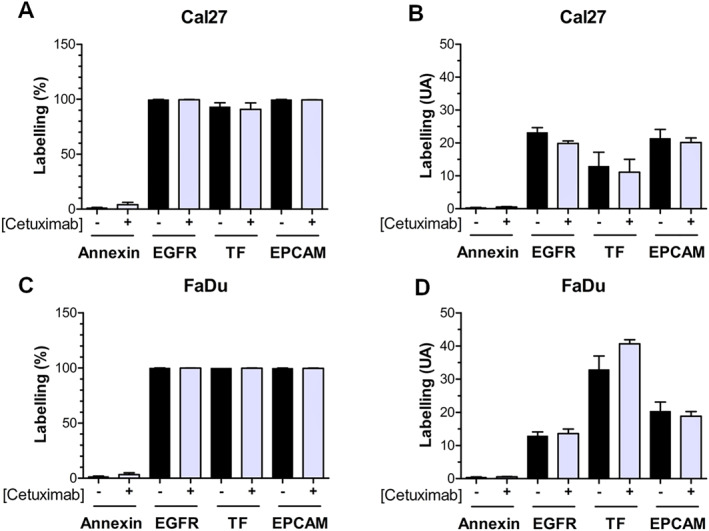
Marker expression by head and neck squamous cell carcinoma cells analyzed by flow cytometry. The binding of annexinV‐APC, anti‐EGFR‐FITC, anti‐TF‐PE, and anti‐EpCAM‐PerCPCy5.5 antibodies at the surface of Cal27 (A and B) and FaDu cells (C and D) was quantified by flow cytometry (Navios). Results were given as labels expressed in the percentage of positive cells (%; A and C) and mean fluorescence intensity in the arbitrary unit (UA; B and D). Differences are considered as significant when *p* < 0.05 using the Bonferroni/Dunn test. * versus the same group without cetuximab, *n* = 3 in duplicate.

**FIGURE 3:**
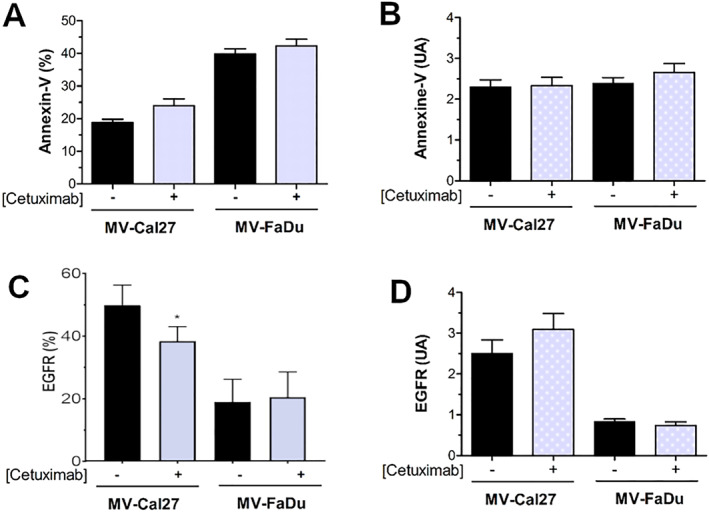
Analysis of the expression of surface marker microvesicles (MVs) by flow cytometry. The binding of annexinV‐APC (A and B) or anti‐EGFR‐FITC (C and D) and anti‐EpCAM‐PerCPCy5.5 antibodies at the MVs surface was quantified by flow cytometry (Navios, Beckman Coulter). Results were given as the percentage of labeling MVs (A and C) and the fluorescence intensity of labeling (B and D). Differences are considered as significant when *p* < 0.05 using the Bonferroni/Dunn test. * versus the same group without cetuximab, *n* = 3 in duplicate.

Both Cal27 and FaDu cell lines expressed surface markers such as EGFR, tissue factor (TF), or EPCAM. Cetuximab treatment did not alter the proportion of cells expressing these markers or their expression levels (Figure 2). It is well established that MVs originate from cells exposing phosphatidylserine (PS) on the outer leaflet of the plasma membrane, a process leading to vesicle budding and fission. Thus, we assessed PS exposure on MVs by annexinV binding using flow cytometry.^17^ Only 20% of MVs from Cal27 or 40% from FaDu bound annexinV‐APC (Figure 3A,B). Cetuximab treatment did not significantly affect annexinV binding of MVs in either cell line. Usually, the protein content of MVs is dependent on their cellular origin. EGFR and EPCAM, both membrane‐associated over‐expressed in HNSCC, were analyzed on the MVs surface. Flow cytometry revealed the presence of EGFR on a subpopulation of MVs (Figure 3C,D), whereas EPCAM was not detected (data not shown). A higher proportion of EGFR‐positive MVs was found in Cal27‐derived MVs compared to FaDu‐derived MVs (Figure 3C, 49% vs. 19% respectively). Cetuximab significantly reduced the proportion of EGFR‐positive MVs from Cal27 (from 49% to 38%) but had no effect on FaDu‐derived MVs (19% vs. 20%). Notably, cetuximab treatment decreased EGFR fluorescence intensity on Cal27 cells (Figure 2B), whereas MVs fluorescence intensity remained unaffected.

### 3.3 Changes in MVs protein content after cetuximab exposure

**FIGURE 4:**
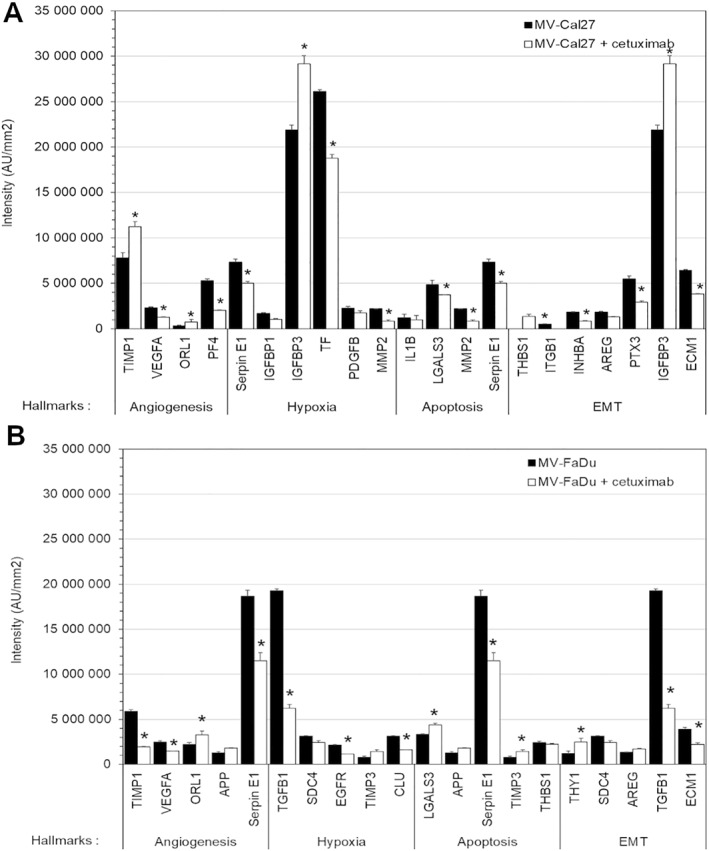
Change of the protein content of microvesicles (MVs) from CAL27 and FaDu cells. The protein content of MVs was evaluated in the lysate extract of purified MVs shedded by Cal27 cells (A) or FaDu cells (B) after exposition of 0–20 μg/mL of cetuximab using human proteome arrays and a change of more than 20% protein is represented. Results were given as intensity/mm^2^ of spots obtained after chemiluminescent detection for each protein found in hallmarks of angiogenesis, apoptosis, hypoxia, and epithelial to mesenchymal transition (EMT). Differences are considered as significant when *p* < 0.05 using the Bonferroni/Dunn test. * versus MV without cetuximab, *n* = 3 in duplicate.

**FIGURE 5:**
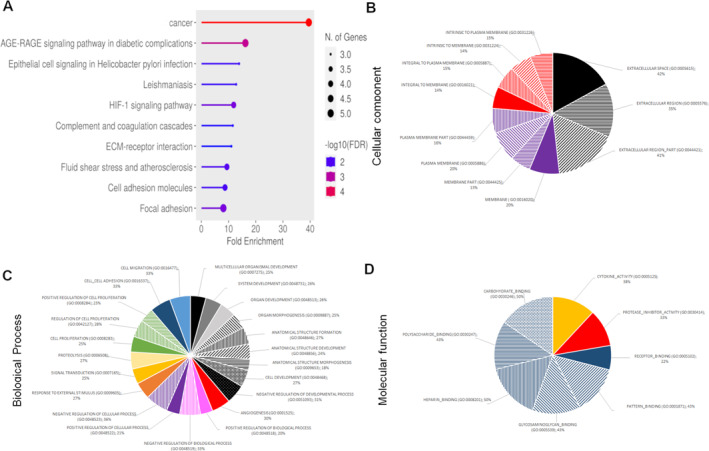
Computational analysis of the protein content of microvesicles (MVs) of CAL27 cells after exposition to cetuximab. MVs protein changes of more than 20% were analyzed after cetuximab exposure of parental cells using gene set enrichment analysis software (GSEA). Signaling pathway (A), cellular component (B), biological process (C), and molecular function (D) were found using the molecular signatures database (MSigDB).

**FIGURE 6:**
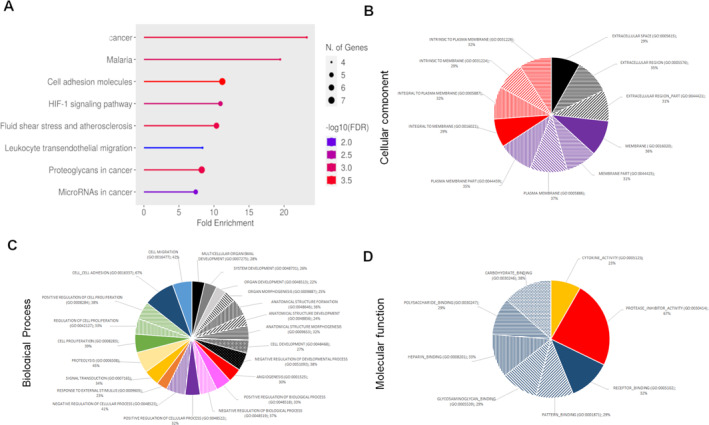
Computational analysis of the protein content of microvesicles (MVs) of FaDu cells after exposition to cetuximab. MVs protein changes of more than 20% were analyzed after cetuximab exposure of parental cells using gene set enrichment analysis software (GSEA). Signaling pathway (A), cellular component (B), biological process (C), and molecular function (D) were found using the molecular signatures database (MSigDB).

Beyond known tumor markers, we investigated whether cetuximab altered the protein content of MVs. Using proteome profiler^TM^ antibody arrays, we screened for 119 human soluble receptors and related proteins and 55 angiogenesis‐related proteins in MVs protein extracts (see Supporting Information S1: S2). Cetuximab altered the MVs protein content, affecting key cancer‐related pathways including inflammation, apoptosis, hypoxia, angiogenesis, or EMT (Figure 4A,B). We selected proteins with expression changes >20% upon cetuximab treatment (Supporting Information S1: S3) and performed a computational analysis to identify the biological effect of cetuximab on the functions of MVs. Using gene set enrichment analysis software, we have explored the molecular signatures database (MSigDB) and find information about signaling pathway (Figures 5A and 6A), cellular component (Figures 5B and 6B), biological process (Figures 5C and 6C), and molecular function (Figures 5D and 6D) of these proteins. The cellular components of these proteins were found close to the plasma membrane and the extracellular region, with limited involvement in complex formation with macromolecules or receptors. Their biological processes were involved in part in development, morphogenesis, and angiogenesis for the two HNSCC cell lines. This effect on angiogenesis affects proteins involved in the formation of vessels but not the protein involved in the regulation of these processes, except for PF‐4 (CXCL4). Biological processes found were also implicated in the response to external stimulus and in the regulation of biological and cellular processes, particularly in signal transduction, proliferation, adhesion, and migration. A great number of proteins implicated in these biological processes were decreased in MVs, but proteins involved in proteolysis were significantly increased. The molecular functions of these proteins were to interact with carbohydrates such as proteoglycan or heparin and also to act on cytokine activity and the inhibition of protease activity. All of these results suggested that cetuximab induces a modification of the MVs content in protein implicated in several process such as angiogenesis, cell proliferation, and migration but increases the content of protein implicated in proteolysis. Most of these proteins decreased significantly in MVs after cetuximab exposure of parental cells.

### 3.4 MVs interaction with endothelial cells and angiogenic potential

As MVs can influence recipient cell behavior, we assessed their ability to interact with ECs by fluorescence‐based microscopic imaging using DiO‐labeled MVs.

**FIGURE 7:**
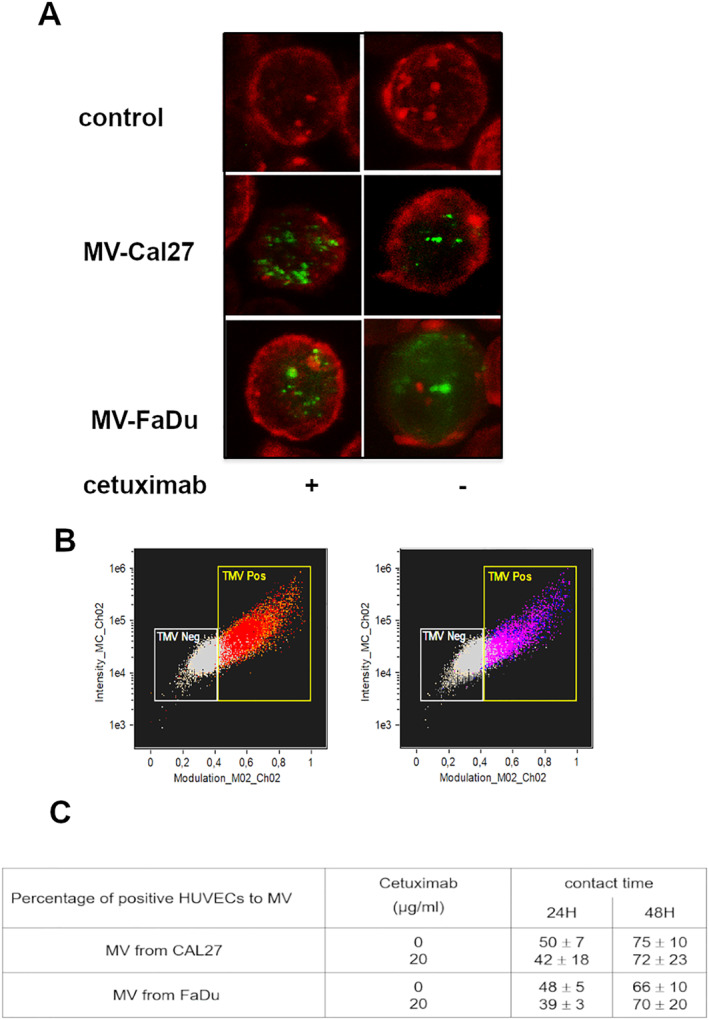
Interaction of microvesicles (MVs) from head and neck squamous cell carcinoma cells with endothelial cells (ECs). MVs obtained from Cal27 or FaDu exposed to 0–20 μg/mL of cetuximab labeled with DiO (green) were incubated 48 h with human umbilical vein endothelial cells (HUVECs) labeled with anti‐PECAM‐APC antibodies (red) to evaluate their internalization by confocal microscopy. The quantification of the interaction of MVs with ECs was analyzed by image flow cytometry (B). The percentage of MV‐positive HUVEC was calculated using the biparametric graph of intensity versus modulation of Channel 2 and the percentage of positive HUVEC to MV following the time of incubation (24 h or 48 h) was represented (C). Differences are considered as significant when *p* < 0.05 using Bonferroni/Dunn test. * versus same group without cetuximab, *n* = 3 in duplicate.

HUVECs were cultured in the presence of CM obtained from DiO‐labeled HNSCC (MV‐DiO). After 24 or 48 h of incubation, the membrane and/or nucleus of ECs was stained using anti‐PECAM‐APC antibody and DAPI, respectively, and observed by confocal microscopy. When cells were cultured in the presence of MV‐DiO, green fluorescent signals corresponding to the DiO dye were observed as circular spots within the cytoplasm of ECs (Figure 7A).

Then, to evaluate the importance of this staining, we have used a fast and high‐speed imaging flow cytometry technique that combines the statistical power of standard flow cytometry with the imaging capability of a digital microscope. As observed for confocal microscopy, a DiO fluorescence signal was also found in HUVEC cultivated in the presence of DiO‐labeled MVs. Moreover, the number of DiO‐positive cells was the same whatever the cellular origin of MVs (50% and 42% for Cal27 and FaDu respectively) and increased with the time of incubation (≈50% vs. ≈75% for 24 and 48 h, respectively) (Figure 7B,C). Furthermore, the number of spots found into the cytoplasm of ECs was calculated using the spot count function of the software. The median number of MV per cell was 4.5, unaffected by treatment or the incubation time (data not shown). Co‐localization analysis confirmed MVs remained cytoplasmic and were not detected in the nucleus (data not shown).

### 3.5 Angiogenic effects of MVs from cetuximab‐treated HNSCC cells

**FIGURE 8:**
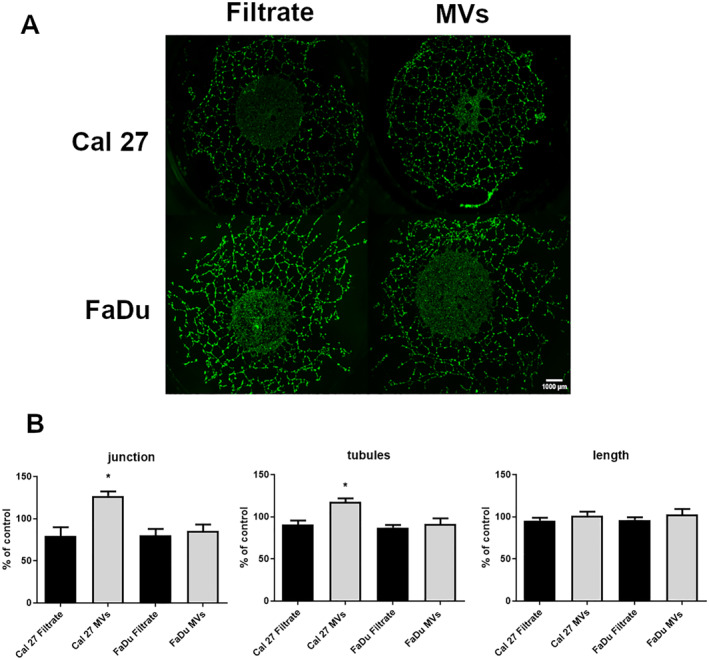
Quantification of the angiogenic parameters after microvesicles (MVs) incorporation. Human umbilical vein endothelial cells seeded on basement membrane Matrigel^TM^ were cultivated in the presence of MVs or filtrates (contained only the soluble factors) obtained from Cal27 or FaDu exposed to 20 μg/mL of cetuximab. Representative images of angiogenic of MVs are presented (A), scale bar = 1000 μm. Junction number and tubule number as well as the total tubule length (B) were quantified by AngioSys software (TCS Cellworks). The values are expressed as the percentage of variation from control media. Differences are considered as significant when *p* < 0.05 using the Bonferroni/Dunn test. * versus the same group without cetuximab, *n* = 3 in duplicate.

To evaluate the angiogenic potential of MVs, HUVECs were cultured on Matrigel™ in the presence of MVs derived from HNSCC cells treated or not to cetuximab (Figure 8A). Some characteristic parameters of the EC network such as the number of junctions (Figure 8B) or tubules (Figure 8C) as well as the length of tubules (Figure 8D) were assessed. Although overall effects were modest, a significant increase of approximately 30% of the number of junctions and 15% of the number of tubules was observed with MV cetuximab‐treated Cal27 cells compared to soluble factors alone (filtered medium condition). These findings suggest that cetuximab exposure may alter MVs composition in a way that subtly affects endothelial organization.

## 4 DISCUSSION

Several lines of evidence have demonstrated a significant role for MVs in tumor progression such as cell growth or angiogenesis.^18^, ^19^ Indeed, MVs can interact with various cells types by fusing with the plasma membrane of target cells and transferring their cargo.^20^ The biogenesis of MVs is often triggered by cellular stress and can potentially be induced by therapeutic agents. However, few studies have investigated how treatment influences MV shedding, content and their subsequent effects on the tumor microenvironment as ECs.

MVs are already considered potential cancer biomarkers and have been identified in the serum or saliva of patient with head and neck cancer.^21^, ^22^ Overexpression of EGFR plays a key role in HNSCC, supporting the use of cetuximab, a monoclonal anti‐EGFR antibody, as well as EGFR‐tyrosine kinase inhibitors (EGFR‐TKI), which inhibit tumor cell proliferation and the secretion of pro‐angiogenic factors by tumor cells, such as VEGF and IL‐8. Additionally, vessel normalization induced by these therapies suggests more complex mechanisms of EC growth regulation in vivo. Our previous work showed that a reduction in VEGF secretion by HNSCC cells exposed to cetuximab may not fully account for the anti‐angiogenic activity in vitro.^15^ In this study, we investigated whether MVs could serve as surrogate biomarkers for the response to anti‐EGFR therapy such as cetuximab. This is particularly relevant in the context of targeted therapies for which reliable surrogate markers of efficacy are needed.^23^ EVs have been shown to interfere with antibody therapy in human epidermal growth factor receptor‐2 (HER‐2) overexpressing breast cancers, where the removal of HER‐2‐positive EVs was proposed to improve therapeutic outcomes.^24^ In our HNSCC models, cetuximab reduced MVs shedding similarly across the different cell lines used. A mechanism involving the internalization of plasma membrane fragments following cetuximab binding to EGFR, as described by Wheeler et al. in 2008,^25^ could explain this decrease. However, a reduction in EGFR surface expression was observed only in Cal27 cells, which are more sensitive to cetuximab than FaDu cells.^16^ MVs formation is known to be regulated by the activation of cell surface receptors with a subsequent increase in intracellular calcium as observed with EGFR.^26^ Therefore, MVs quantification could potentially serve as an indicator of the response to cetuximab, although the specific identification of tumor‐derived MVs in biological fluids remains a challenge. Since MVs are released upon cell activation and exposure of phosphatidyl‐serine on the outer leaflet of the plasma membrane, annexinV binding is widely used for MVs identification. However, we found that only about half of MVs from HNSCC cells bound annexinV. This may be due to membrane flip‐flop during MVs release or sample preparation, despite minimal sample handling and the absence of freezing or cooling. Notably, PS exposure and MVs release can be uncoupled in some contexts,^27^ suggesting that PS‐negative MVs may escape detection by annexinV and challenge the reliability of this method. Identifying tumor‐derived MVs through surface proteins is a promising alternative, as various tumor membrane proteins may be selectively incorporated into MVs membranes during biogenesis. To explore this, we screened 155 proteins using proteome arrays. Cetuximab appeared to reduce the total protein content of MVs, decreasing the expression of most receptors tested. However, galectin‐3BP and lipocalin‐2 levels increased in MVs from Cal27 cells. These two receptors are involved in the immune response; galectin‐3BP functions as cell to cell or matrix interaction and lipocalin‐2 can act as a growth factor. Surprisingly, EpCAM, which has been yet used to isolate EVs from the human colon or ovarian carcinoma cells ^17^, was not detected on MVs from HNSCC cells. We evaluated also EGFR expression on the MVs surface, as EGFR is a common cancer biomarker previously detected on the cancer cells surface.^28^ Notably, cetuximab significantly reduced EGFR‐positive MVs only in the cetuximab‐sensitive cell line Cal27. Taken together, these results suggest that cetuximab differentially affects MVs content and surface expression depending on the cell type. Membrane receptors may be selectively incorporated within or excluded from MVs, and EGFR surface expression on MVs could serve as a potential marker of cetuximab efficacy. Moreover, EGFR inhibitors may limit HNSCC malignancy not only through the inhibition of EGFR signaling but also by impairing exosome‐mediated communication via macropinocytosis ^29^

Given that cetuximab alters the MVs protein content, it is important to assess how the changes affect the recipient cells in the tumor microenvironment cells, especially ECs. The ability of MVs to influence angiogenesis has been previously demonstrated.^19^, ^30^ A previous work showed that MVs from tumor cells can induce a transformed phenotype in ECs.^20^ Although the exact mechanisms of MV–EC interactions are still unclear, plasma membrane protein binding appears to be involved. In our experiments, approximately 50% of ECs internalized DiO‐labeled MVs after 24 h of incubation, with this number increasing over time. However, DiO labeling efficiency was limited to about 60%, likely due to MVs membrane rearrangement during sample preparation. This labeling was sufficient to assess MV–EC interactions. Cetuximab did not alter MVs internalization by ECs. However, it modified the angiogenic protein cargo of MVs, decreasing pro‐angiogenic factors like TF, PAI‐1, or uPA and increasing anti‐angiogenic proteins such as endostatin and TIMP‐1. Interestingly, despite this anti‐angiogenic shift in MVs content, MVs from cetuximab‐treated Cal27 cells still promoted angiogenesis, consistent with effects observed for CM from the same cells.^16^ However, key pro‐angiogenic factors such as IL‐8 and ANG1 were absent or weakly present as VEGF in MVs, although they were detectable in the secretome of Cal 27 and FaDu cells.^16^ Despite the protein richness of MVs,^31^ they can also carry nucleic acid that may influence EC behavior.^32^ Therefore, even though cetuximab reduces MVs release, the altered MVs cargo may contribute to resistance mechanisms and should be evaluated independently from soluble factors.

In conclusion, MVs may serve as surrogate biomarkers to predict the efficacy of anti‐EGFR therapies, provided EGFR can be reliably detected on their surface. Furthermore, MVs release following treatment should be considered as a potential resistance mechanism and carefully monitored to better assess the therapeutic impact on the tumor microenvironment.

## AUTHOR CONTRIBUTIONS

V. J.‐H. and C. B. designed research; V. J.‐H. performed research; V. J.‐H., C. B. and B. F. analyzed data; V. J.‐H., C. B. and B. F. wrote paper and B. F. was guarantor of the study.

## CONFLICT OF INTEREST STATEMENT

The authors declare no conflicts of interest.

## ETHICS STATEMENT

None.

## Supporting information

Supporting Information S1

## Data Availability

The datasets used or analyzed during this study are available from the corresponding author upon reasonable request.
