# Supplementary material for: Cetuximab modifies the release and protein content of tumor microvesicles from head and neck squamous cell carcinoma cells: What are the consequences on endothelial cells?
Source: J Cell Commun Signal. 2025 Aug 4;19(3):e70026. doi: 10.1002/ccs3.70026 (PMC12321591; doi:10.1002/ccs3.70026)
Supplement: Supplementary file 1 — Supporting Information S1 [file CCS3-19-e70026-s001.docx]

**Supplementary Data**

**S1: Quantification of the diameter of microvesicles released into the conditioned media**

Megamix beads were used to build a calibration curve in order to connect the measured value of forward side scatter (FSC) with the theoretic size of these beads. Thus the diameter of each MV was calculated using linear regression equation of the calibration curve and the size distribution of the MVs population was presented as a histogram for each condition (CM-Cal27 or CM-FaDu for HNSCC exposed or not to 20 µg/ml of cetuximab). Median diameter of MVs shedded by HNSCC were given for each condition with standard deviation (SD).

**A**


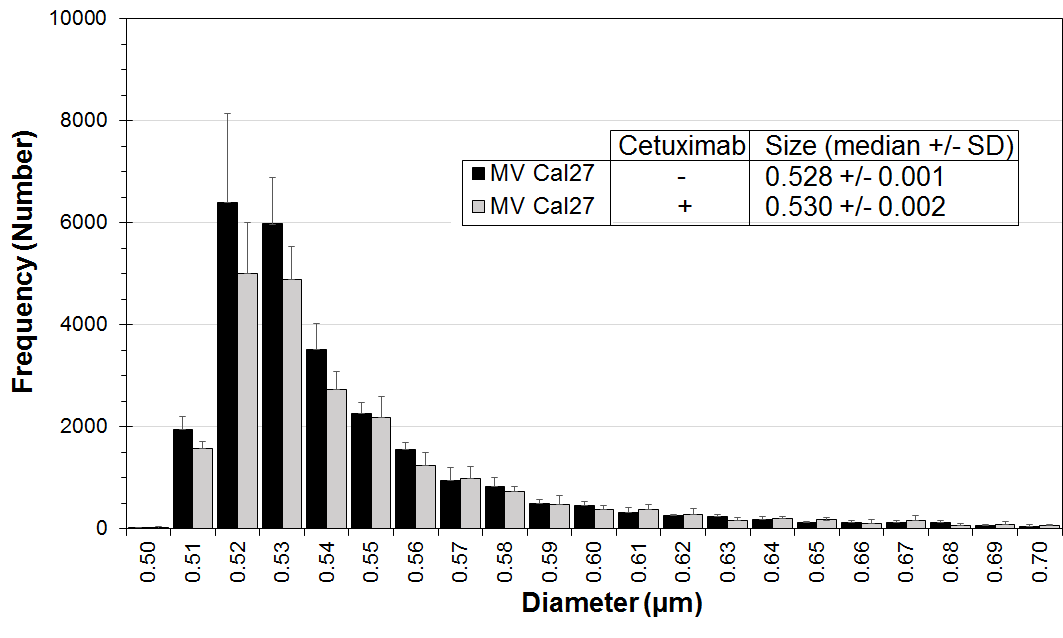


**B**


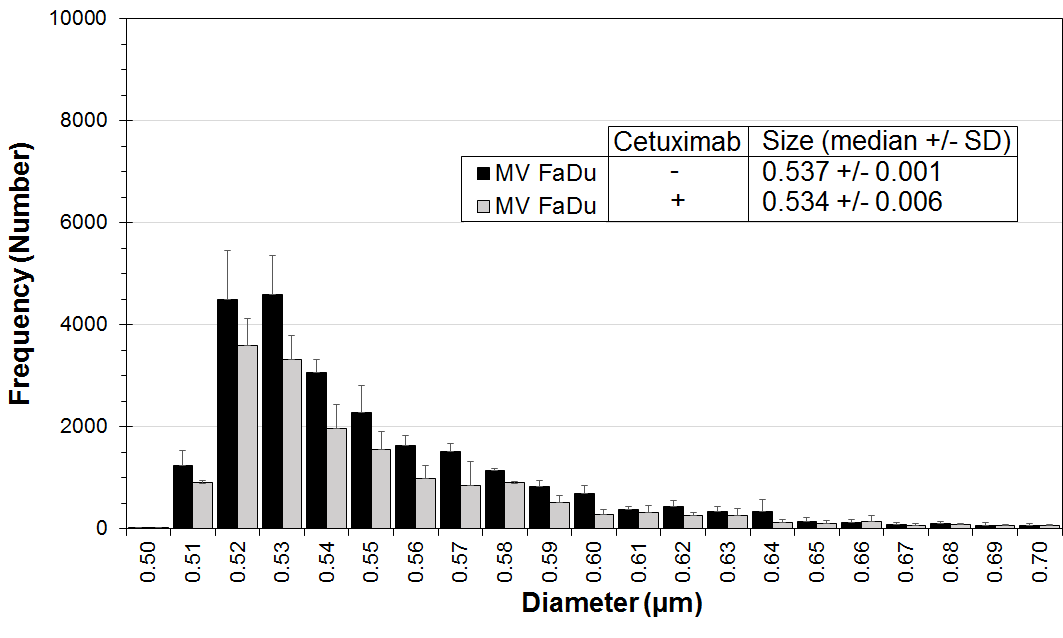


**S2: List of all proteins detected in proteome profiler antibody array kit (bio-techne/R&D System)**

**Human angiogenesis array** (ARY007; 55 angiogenesis related proteins)

| Activin A | FGF-7/KGF | PD-ECGF |
| --- | --- | --- |
| ADAMTS-1 | GDNF | PDGF-AA |
| Angiogenin | GM-CSF | PDGF-AB/PDGF-BB |
| Angiopoietin-1 | HB-EGF | Persephin |
| Angiopoietin-2 | HGF | CXCL4/PF4 |
| Angiostatin/Plasminogen | IGFBP-1 | PlGF |
| Amphiregulin | IGFBP-2 | Prolactin |
| Artemin | IGFBP-3 | Serpin B5/Maspin |
| Tissue Factor/Factor III | IL-1 beta | Serpin E1/PAI-1 |
| CXCL16 | CXCL8/IL-8 | Serpin F1/PEDF |
| DPPIV/CD26 | LAP (TGF-beta 1) | TIMP-1 |
| EGF | Leptin | TIMP-4 |
| EG-VEGF | CCL2/MCP-1 | Thrombospondin-1 |
| Endoglin/CD105 | CCL3/MIP-1 alpha | Thrombospondin-2 |
| Endostatin/Collagen XVIII | MMP-8 | uPA |
| Endothelin-1 | MMP-9 | Vasohibin |
| FGF acidic | NRG1-beta 1 | VEGF |
| FGF basic | Pentraxin 3 | VEGF-C |
| FGF-4 |  |  |

**Human Soluble Receptor Array** (ARY 012; 119 soluble receptors and related proteins)

**→ Non-Hematopoeitic Array**

| ADAM15 | Integrin alpha 6/CD49f |
| --- | --- |
| betaIG-H3 | Integrin alpha 9 |
| BMPR-IB/ALK-6 | Integrin alpha V/CD51 |
| Cadherin-4/R-Cadherin | Jagged 1/CD339 |
| Cadherin-11 | JAM-B/VE-JAM/JAM2/CD322 |
| Cadherin-13 | JAM-C/JAM3 |
| E-Cadherin | LRP-6 |
| N-Cadherin | MCAM/CD146 |
| P-Cadherin | MEPE |
| VE-Cadherin | MUCDHL |
| Cathepsin D | Nectin-2/CD112 |
| CD40 | Nectin-4 |
| CEACAM-5/CD66e | Neurotrimin |
| CHL-1/L1CAM-2 | Notch-1 |
| Clusterin | NrCAM |
| Coagulation Factor II/Thrombin | Periostin/OSF-2 |
| COMP/Thrombospondin-5 | Podocalyxin |
| CRELD2 | E-Selectin |
| Desmoglein 2 | Semaphorin 3A |
| ECM-1 | SREC-I/SR-F1 |
| EGF R/ErbB1/HER1 | SREC-II |
| Endoglycan | Stanniocalcin 1 |
| EpCAM/TROP-1 | Syndecan-1/CD138 |
| ErbB2/HER2 | Syndecan-4 |
| ErbB3/HER3 | Thrombospondin-2 |
| ErbB4/HER4 | TIMP-4 |
| ESAM | TROP-2 |
| Galectin-2 | VAP-1/AOC3 |
| HPRG | VCAM-1 |
| Integrin alpha 3/CD49c | VEGF R1/Flt-1 |
| Integrin alpha 5/CD49e | VEGF R2/KDR/Flk-1 |

| **→ Common Analytes Array** | |
| --- | --- |
| ACE | HB-EGF |
| ADAM8 | ICAM-2/CD102 |
| ADAM9 | IL-1 RII |
| ADAM10 | IL-15 R alpha |
| ALCAM/CD166 | Integrin beta 1/CD29 |
| Amphiregulin | Integrin beta 2/CD18 |
| APP (pan) | Integrin beta 3/CD61 |
| BACE-1 | Integrin beta 4/CD104 |
| BCAM/CD239 | Integrin beta 5 |
| C1q R1/CD93 | Integrin beta 6 |
| CD9 | JAM-A/CD321 |
| CD23/Fc epsilon RII | Lipocalin-2/NGAL |
| CD31/PECAM-1 | LOX-1/SR-E1/CLEC8A |
| CD36/SR-B3 | MD-1/LY86 |
| CD40 Ligand | MMP-2 (total) |
| CD44H | NCAM-1/CD56 |
| CD58/LFA-3 | NCAM-L1/CD171 |
| CD90/Thy1 | Osteopontin |
| CD99 | PAR1 |
| CD155/PVR | Pref-1/DLK-1/FA1 |
| CEACAM-1/CD66a | RECK |
| CX3CL1/Fractalkine | Stabilin-1 |
| CXCL8/IL-8 | TACE/ADAM17 |
| EMMPRIN/CD147 | Thrombospondin-1 |
| Endoglin/CD105 | TIMP-1 |
| Epiregulin | TIMP-2 |
| Galectin-1 | TIMP-3 |
| Galectin-3 | TNF RII |
| Galectin-3BP/MAC-2BP |  |

**S3: Percentage of variation of protein content in MVs after cetuximab exposition**
